# Knowledge, Attitudes and Practices of Parents and Pediatricians Regarding Antibiotic Use among Children: Differences in Relation to the Level of Education of the Parents in the Republic of Srpska Bosnia and Herzegovina

**DOI:** 10.3390/antibiotics11101325

**Published:** 2022-09-28

**Authors:** Biljana Mijović, Jela Aćimović, Jelena Đaković Dević, Julija Kralj, Vesna Lučić Samardžija, Mirjana Djermanović, Marija Milić, Vesna Vujić-Aleksić, Snežana Perić Simić, Bojan Joksimović

**Affiliations:** 1 Faculty of Medicine Foča, University of East Sarajevo, 73 300 Foča, The Republic of Srpska, Bosnia and Herzegovina; 2 Public Health Institute of The Republic of Srpska, 78 000 Banja Luka, The Republic of Srpska, Bosnia and Herzegovina; 3 Faculty of Medicine, University of Banja Luka, 78 000 Banja Luka, The Republic of Srpska, Bosnia and Herzegovina; 4 Primary Healthcare Centre, 78 000 Banja Luka, The Republic of Srpska, Bosnia and Herzegovina; 5 Department of Epidemiology, Faculty of Medicine, University of Pristina Temporarily Settled in Kosovska Mitrovica, 38 220 Kosovska Mitrovica, Serbia; 6 Agency for Certification, Accreditation and Quality Improvement in Health Care of The Republic of Srpska, 78 000 Banja Luka, The Republic of Srpska, Bosnia and Herzegovina; 7 Primary Healthcare Centre, 76 300 Bijeljina, The Republic of Srpska, Bosnia and Herzegovina

**Keywords:** outpatient antibiotic consumption, COVID-19, antimicrobial management, mortality

## Abstract

Antibiotics are often misused, especially for the treatment of upper respiratory tract infections (URTIs) in children, where their use is unnecessary and leads to antimicrobial resistance. This study sought to explore the knowledge, attitudes and practices (KAP) of parents and pediatricians on the use of antibiotics among children and whether the level of education of parents has an impact on their KAP. The research was carried out among 1459 parents of children under 6 years of age and among 18 pediatricians. Sixty percent of pediatricians (61.1%) were prescribed antibiotics daily in their practice. Most of the surveyed parents (98.4%) state that doctors are their main source of information when deciding on the use of antibiotics in the treatment of their children. Parents with a higher level of education use television less often as a source of information when making this decision compared to parents with a lower level of education (*p* = 0.039, i.e., *p* = 0.003). The majority of parents (80.7%) knew that Panklav (amoxicillin/clavulanic acid) is an antibiotic, while 52.5% identified Pancef (cefixime) as an antibiotic. Parents with a higher level of education correctly identified antibiotics significantly more often (*p* < 0.001). This study shows that in the Republic of Srpska, parents have adequate knowledge about antibiotics, especially those with a higher level of education, who show better KAP when it comes to antibiotic use.

## 1. Introduction

The greatest medical achievement of the 20th century was the introduction of antibiotics into clinical practice [1], and their proper use reduces morbidity and mortality [2]. Except in treating many infectious diseases, antibiotics have made many modern medical procedures possible, including organ transplants, cancer treatment and prevention of infections in surgeries [3]. However, antibiotics are often misused, especially for the treatment of upper respiratory tract infections (URTIs) in children, where the use of antibiotics is unnecessary and not suitable. However, there is enough evidence of the predominantly viral origin of these conditions and proof that this practice does not influence the severity of symptoms and duration of the disease [4,5]. Although the misuse and overuse of antibiotics increase the cost of health services, the frequency of side effects such as diarrhea leads to antibacterial resistance, due to which some infections are not treated today. The consumption of antibiotics in the world is constantly increasing [3,5,6]. Knezevic et al., in their retrograde analysis of the laboratory documentation at the University Clinical Center of the Republic of Srpska (RS), identified a high prevalence of multidrug resistance *Acinetobacter* spp., especially in the intensive care unit (ICU) [7]. The isolates were more resistant in the ICU than isolates originating from the surgical, internal and gynecological clinic [7]. The report by O’Neill, commissioned by the United Kingdom Government, predicted that without urgent measures, more than 10 million people will die annually from drug-resistant infections by 2050 [8]. The strong connection between antibiotic excessive use and the development of antimicrobial resistance has already been proven, and many countries with high antibiotic consumption have reported a high prevalence of resistant bacteria [9,10]. As a result, the World Health Organization (WHO) recognized that the emergence of antibiotic resistance is one of the top 10 public health threats facing humanity on a global level [11].Furthermore, multiple studies have reported a sudden high rate of utilization of antibiotics during the coronavirus disease-19 (COVID-19) pandemic [12,13,14]. This practice led to a higher incidence of infections due to methicillin-resistant *S. aureus* and carbapenem-resistant *Acinetobacter baumannii* among patients with COVID-19. The authors suggested that in order to mitigate the long-term impact of COVID-19 on antimicrobial resistance, the integration of new antimicrobial stewardship activities is needed, not just in the hospital but also in the outpatient setting [15].

Based on numerous reports, there are many complex reasons for the unnecessary and inappropriate use of antibiotics in children. This may be due to the fact that in families, when common infections occur in children, previously used antibiotics familiar to parents are often used for treatment without consulting a pediatrician [16]. Additional factors leading to antibiotic overuse in children are overprescribing by doctors due to diagnostic insecurity [17], easy access to antibiotics for self-medication [18], and very often pressure on pediatricians to prescribe antibiotics due to lack of knowledge and low level of education of parents and their indifference [19,20]. Studies from European, American and Asian countries indicate that many parents (22% and 70%)have the wrong conception about the appropriate use and efficacy of antibiotics, and often use them without prescription [21,22]. As a result, treatment with antibiotics provides minimal benefit, and even harm (many side effects, antibiotic resistance, etc.) [23,24].

The RS is one of the two entities of Bosnia and Herzegovina. The RS has implemented many activities to regulate enhancing the appropriate use of antibiotics, including the use of good clinical practice guidelines [25,26]. The policy of restricting the excessive use of antibiotics in the RS includes restrictions on the sale of antibiotics, where a pharmacist can dispense antibiotics only on a doctor’s prescription [26].These measures significantly increased prescribing efficacy in accordance with standard clinical treatment guidelines, positively impacting the rate of polypharmacy in clinical practice [27,28]. Even though Bojanic et al., in their observational retrospective study of total outpatient antibiotic consumption in the RS, addressed some concerns with the consumption of amoxicillin/clavulanic acid and azithromycin, they concluded that multiple recent interventions in the RS resulted in one of the lowest consumption rates of antibiotics when compared with similar countries in the Balkan region [26].

Bajcetic and Jovanović observed a significant lack of knowledge and skills in administering antibiotics in children by parents. The authors suggested that parents lack basic and essential knowledge to deal with the antibiotics they administer to their children [29]. Because of wide cross-national significant differences in the use of antibiotics [30], and the lack of knowledge and education of parents about antibiotic use among children [21,22,29], which is the cause of misuse [20], this study sought to explore the knowledge, attitudes and practices of parents and pediatricians on the use of antibiotics among children, and whether the level of education of parents has an impact on their knowledge, attitudes and practice when it comes to antibiotic usage. This could serve as possible baseline data and provide further insight into developing and planning strategies for regional and national education purposes about the rational use of antibiotics among children.

## 2. Results

Out of a total of 1459 respondents, the majority of the surveyed parents were female (75.5%). The average age of the respondents was 34.47 ± 6.15 years. The youngest respondent was 16 years old, and the oldest was 59 years old. The majority of respondents (82.3) lived in urban areas. The largest number of mothers (49.3%) had a university degree, while 43.1% reported having completed high school. The largest number of fathers (54.1%) had completed high school, while 37.1% of fathers had a university degree. The largest percentage of respondents (81.5%) rated their financial income as average. The majority of respondents (63.7%) have a professional relationship with their child’s pediatrician; 34.3% believe that they have a friendly relationship, while 2% of respondents state that their relationship with the pediatrician is familiar. Sixty-seven respondents (4.6%) were single parents. The largest number of parents (47.8%) had two children, 32.3% had one child, 16.2% of parents had three children, 2.7% of parents had four children, while only six parents (0.4%) had five children (Table 1).

Out of a total of 18 pediatric specialist respondents in the RS, 72.2% were female; the largest number of them were aged 56 to 65 (61.1%);and 22.2% had work experience of 3 to 10 years, 27.8% from 16 to 20 years, 22.2% from 21 to 25 years, while 27.8% of pediatricians had more than 25 years of experience. All surveyed pediatricians (100%) believe that their work contributes to the fight against antibiotic resistance, all (100%) know what antibiotic resistance is, 77.8% know what information to provide patients about the rational use of antibiotics, and 72.2% believe that they have sufficient knowledge of how to use antibiotics in their practice. All pediatricians (100%) know that antibiotics are not effective in the treatment of colds and flu, 100% of them know that the use of antibiotics in therapy is associated with side effects and risks such as diarrhea, colitis and allergies, while 94.4% know that any person treated with antibiotics has an increased risk of developing an infection that is resistant to antibiotics. In addition, all pediatricians (100%) know that healthy people can be carriers of bacteria that are resistant to antibiotics (Table 2).

Examining the attitudes of pediatricians and their practice when it comes to the use of antibiotics in treatment, it was determined that 61.1% of pediatricians in the last 12 months received information about the importance of avoiding the unnecessary prescription of antibiotics in treatment. Not a single pediatrician received information at a workplace about the most important influence on the change of understanding in prescribing antibiotics; 44.4% of pediatricians received this information through a clinical practice guide, 11.1% from colleagues, 27.8% from scientific organizations, while not a single pediatrician received this information from social networks. With regard to the received information about the correct use of antibiotics in therapy, only 61.1% of pediatricians stated that they changed the practice of prescribing antibiotics after receiving the information. The largest number of pediatricians (88.9%) believe that the most effective way to combat antibiotic resistance is to take action at all levels, 33.3% believe that it is most effective to initiate action at the individual level would include all healthcare workers, 33.3% believe that global action is necessary at the European Union level, while the smallest number (22.2%) believes that action at the regional or national level is sufficient. More than half of the surveyed pediatricians (55.5%) believe that in the RS, there is good promotion of the rational use of antibiotics and the importance of antibiotic resistance. Sixty percent of pediatricians (61.1%) prescribe antibiotics in their practice every day, 38.9% prescribe antibiotics on a weekly basis, while 66.7% take antibiotic resistance into account when treating patients. A large number of pediatricians stated that they often prescribe antibiotics out of fear of aggravation of patients’ symptoms or risk for complications, 5.6% of pediatricians do it every day, 11.1% less than once a week, 33.3% once a week or more often, and 50% never prescribe antibiotics from fear of complications. The majority of pediatricians (61.1%) rarely prescribe antibiotics due to uncertainty in the diagnosis of infection, 5.6% prescribe antibiotics daily due to uncertainty in the diagnosis, while 33.3% never do so. All pediatricians (100%) use the strategy of educating patients in order to rationally prescribe antibiotics, 11.1% use the strategy of consultation with a new patient, while 55.6% use delayed prescribing (Table 3).

Most of the surveyed parents (98.4%) state that doctors are their main source of information when deciding on the use of antibiotics in the treatment of their children, 2.7% state that television is their main source of information, while for 4.3% of parents, friends are the main source of information when making this decision. No significant difference was observed between the groups of parents divided in relation to the level of education when it comes to doctors as the main source of information when making decisions about the use of antibiotics in children’s therapy. In other words, both lower- and higher-educated parents equally believe that doctors are their main source of information when making a decision. However, it was observed that mothers (1.9%) and fathers (1.2%) with a higher level of education (college or university degree) use television less often as a source of information when making this decision compared to mothers (3.6%) and fathers (3.8%) with a lower level of education (completed primary and high school) (*p* = 0.039, i.e., *p* = 0.003). In addition, mothers (5.9%) and fathers (5.5%) with a lower level of education significantly more often use friends as a source of information when making a decision about the use of antibiotics in their children compared to more educated mothers (3%) and fathers (2, 8%) (*p* = 0.007, i.e., *p* = 0.012) (Table 4).

When asked which of the listed medicines are antibiotics, 80.7% of parents knew that Panklavis an antibiotic, with mothers and fathers with a higher level of education (86.5% i.e., 86.9%) showing significantly better knowledge compared to respondents with a lower level of education (73.7% i.e., 75.8%) (*p* < 0.001, i.e., *p* < 0.001). In addition, a slightly lower percentage of parents knew that Pancefis an antibiotic (52.5%), whereby mothers (62.8%) and fathers (61.8%) with a higher level of education knew significantly more often that Pancef was an antibiotic compared to mothers (40.1%) and fathers (45.2%) with a lower level of education (*p* < 0.001, i.e., *p* < 0.001). Ninety-three percent of respondents knew about paracetamol and 93.6% about ibuprofen that they are not antibiotics, with significantly better knowledge shown by mothers (*p* < 0.001) and fathers (*p* < 0.001) who had a higher level of education compared to mothers and fathers with a lower level of education (Table 5).

The majority of surveyed parents (82.3%) do not agree with the statement that antibiotics should be given to every child who has a fever, 10.6% have a neutral attitude, while 7% of respondents agree with this statement. Mothers (90.7%) and fathers (89.8%) with a higher level of education disagree with this statement in a significantly greater number than mothers (72.3%; *p* < 0.001) and fathers (76.6%; *p* < 0.001) with a lower level of education. Twenty-six percent of respondents (26.3%) disagree with the statement that scientists can easily produce new antibiotics that could destroy resistant bacteria, 39.5% have a neutral attitude, while 34.2% of respondents agree with this statement. In this case, too, more educated mothers (31.3%) and fathers (29.5%) disagree with this statement in greater numbers than mothers (20.3%; *p* < 0.001) and fathers (23.9%; *p* < 0.001) who have a lower level of education. Seventy-five percent (74.8%) of parents believe that antibiotics can cause side effects; significantly more often this opinion is held by mothers (79.1%) and fathers (79.1%) with a higher level of education compared to mothers (69.4%; *p* < 0.001) and fathers (71.2%; *p* < 0.001) who had a lower level of education (Table 6).

Examining the reasons why parents give antibiotics to their children without a pediatrician’s recommendation, it was determined that 2.2% of parents often or always give antibiotics to a child without a pediatrician’s recommendation due to lack of time to take the child to a pediatrician’s examination or lack of money to pay for the child’s treatment. This is done significantly more often by mothers (1.5%) and fathers (1.1%) who have a lower level of education compared to mothers (0.4%; *p* < 0.001) and fathers (0.6%; *p* < 0.001) with a higher level of education. Almost 3% (2.6%) often and 3.1% always give antibiotics to a child without a pediatrician’s recommendation because the pediatrician previously prescribed the same antibiotic for the same symptoms, significantly more often lower educated mothers (5.3%) and fathers (4.3%) compared to more educated mothers (1.4%; *p* < 0.001) and fathers (1.6%; *p* < 0.001). Antibiotics are often given to children by 2% of parents because the pharmacist recommended it, and almost 1.7% of parents do it always, again, more often mothers (3.3%) and fathers (2.1%) with a lower level of education than mothers (0.4%; *p* < 0.001) and fathers (1.2%; *p* < 0.001) with a higher level of education. One percent (1.2%) of respondents often and 1.3% always give antibiotics to their child because a friend or relative recommended it to them. Less educated mothers (1%) and fathers (1.6%) listen to the advice of friends and relatives significantly more often than more educated mothers (0.5%; *p* < 0.001) and fathers (1%; *p* < 0.001) (Table 7).

## 3. Materials and Methods

### 3.1. Design and Study Population

The research was carried out in 2020 among 1459 parents of children under 6 years of age who brought their children to the previously selected primary health care centers and among parents of children under 6 years of age that attended preschool institutions in previously selected municipalities. In addition, 18 pediatricians were included in the research.

According to 2020 estimates, there are 1,136,274 inhabitants in the RS [31].Four cities with urban status (Banja Luka, Bijeljina, Derventa, Istočno Sarajevo) and four municipalities with rural status (Berkovići, Foča, Bileća and Ljubinje) in the RS were randomly selected. Then, in each of these cities, two health care centers and two preschool institutions were randomly selected, and research was conducted in them. Pediatricians were recruited from all health centers in the territory of the selected municipalities.

The cross-sectional study was conducted simultaneously in all selected cities and municipalities and included parents or legal representatives of preschools or primary healthcare centers and pediatric specialists. All respondents agreed to participate in the study and signed informed consent. The study was approved by the Ethics Committee of the Public Health Institute of the RS. Participation was voluntary, and all collected data were encrypted and thus entered into the database.

As a research instrument, structured questionnaires, specially designed for this research, were used. Questionnaires were distributed to parents upon picking up their children from preschools or after leaving health care centers, and questionnaires for pediatricians were distributed to health centers in all mentioned cities and municipalities.

The questionnaires for parents and pediatricians consisted of 44 questions divided into four groups and were developed after reviewing similar published studies [32,33]. It was pretested on 20 parents and 8 pediatricians. The first group of the questionnaire included basic sociodemographic characteristics. The second group included questions about parental and pediatricians’ knowledge about antibiotics. The third group was related to the parental and pediatricians’ knowledge, attitudes and behavior about antibiotic use. The fourth group examined parental and pediatricians’ attitudes towards antibiotic prescribing in pediatric practice.

### 3.2. Statistical Analyses

The methods of descriptive and analytical statistics were used for data description and analysis. Among the methods of descriptive statistics, measures of central tendency and measures of variability were used, namely arithmetic mean with standard deviation. For the univariate analyses of possible differences in frequencies between groups divided by level of education of parents nonparametrically, the Chi-square test was used. The usual value of *p* < 0.05 was taken as the level of statistical significance of differences. All statistical analyses were performed using IBM SPSS Statistics Software version 24.0 for Windows (IBM Co., Armonk, NY, USA).

## 4. Discussion

The current study aimed to analyze the knowledge, attitudes and practices of parents and pediatricians concerning antibiotic use among children, and whether the level of education of parents has an impact on their knowledge, attitudes and practice when it comes to antibiotic usage. The issue of the current study is novel in the RS population.

Our study demonstrated that RS parents and pediatricians have a trusting relationship. A great majority of parents (98.4%) have confidence in the information and prescriptions supplied by pediatricians. Furthermore, most of the parents chose them as the main source of information about the use and misuse of antibiotics. Only 2.7% of our parents chose television as the main source of information regarding antibiotic use, while 4.3% of parents chose friends as a preferred source of information about antibiotic usage. In both cases, parents with a higher educational level significantly less often use television or friends as a source of information when deciding on the use of antibiotics. Despite the fact that the majority of similar studies have reported pediatricians as the preferred source of information, such as studies conducted in Cyprus [19] and Palestine [34], a study in China conducted by Xiang et al. [35] identified television as the main source of information regarding the use or misuse of antibiotics. Studies have shown that education about the rational use of antibiotics is useful in increasing knowledge, but that they have very little effect in changing the attitudes and practices of the public towards the use of antibiotics [36,37]. Different types of interventions aimed at the rational use of antibiotics (educational interventions, theatrical and musical interventions, gamification, animated film, fear-based messages highlighting the side effects of antibiotic use, digital interventions), which until now have been used in the world and mostly target the public, have produced modest results [36]. A study examining the impact of a television campaign on antibiotic use indicated that it was wrong to emphasize that the antibiotic user was responsible for the development of resistance because it was interpreted as childish by viewers. Instead, it is considered that accurate and real information about the magnitude of the problem of antimicrobial resistance and the specific risk of excessive use of antibiotics should be disseminated through television in order to motivate the audience to change attitudes and behavior towards antibiotic use [38]. Adequately trained pharmacists integrated into the health care system can minimize the inappropriate use of antibiotics by supervising the issuing of prescriptions, using repeated prescriptions, adjusting the amount dispensed in relation to the prescribed amount, using pharmacies in campaigns and providing information to promote and raise awareness about the use of antimicrobial drugs, training of pharmacy students, and cooperation with doctors who prescribe drugs [39,40,41]. The majority of parents believe that their families are exposed to a low risk of antibiotic resistance because they are infrequent users of antibiotics and behave morally following the campaign’s messages. They generally point out that future campaigns should have a relevant, accessible message with a focus on outcomes that parents of young children can relate to (e.g., recurrent infection) and in a format that parents will engage with (e.g., parent/child community events), per possibilities of face-to-face messages [42].

Our results demonstrated that 7% of parents agreed with the statement that an antibiotic should be given to every child who has a fever, while all pediatricians (100%) knew that antibiotics are not effective against cold and flu. However, the mothers and fathers with a lower level of education significantly more often agreed with this statement when compared to mothers and fathers who had a higher level of education. A Palestinian study conducted by Zyoud et al. demonstrated that fever symptoms accompanied by URTIs are the most common reason for a pediatric visit, in which many parents would expect to receive antibiotics for treatment [34]. Similarly, a cross-sectional Malaysian study conducted on a sample of 421 parents demonstrated that more than two-thirds (76%) of parents were convinced that antibiotics were helpful as a cure for fever [43]. Our results demonstrated that 74.8% of parents and 100% of pediatricians were well informed that therapy with antibiotics could potentially be harmful with side effects and risks such as diarrhea, colitis and allergy. Significantly more often this opinion was shared by parents with a higher level of education when compared to parents with a lower educational level. However, even though a high number of our parents are aware that antibiotic use is associated with harmful and adverse effects on body organs, this should not mean that they would not use antibiotics on their own, without consultation with pediatricians. In a Palestinian cross-sectional study [34], where 400 parents were questioned. almost 51% of parents choose antibiotics as the first choice for treatment URTIs, and even 78% of them understood that hepatotoxicity and nephrotoxicity are the most common adverse effects of antibiotics. Furthermore, this study showed that 72.7% of questioned parents believed that antibiotics were used unnecessarily, and comparable results were found in similar previous studies by Rouusounides (81%) [19] and Panagakou (78%) [33]. This prescribing policy could be attributed to the pediatrician’s behavior. There are a few factors that may induce inappropriate prescription of antibiotics such as diagnostic uncertainty, lack of knowledge, fear of litigation or social and cultural pressure [44,45]. In our study, 50% of pediatricians stated that they never prescribed antibiotics to a child because of fear of complications or fear of worsening the patient’s condition; however, the other 50% stated that this was the exact reason to prescribe antibiotics at least once a week or less frequently. In addition, 66.6% of our pediatricians once a day or rarely prescribe antibiotics to a child because they were unsure of the diagnosis of an infection. The doctors point out that during their studies, they received little information from their professors about the magnitude of the problem of antimicrobial resistance and the rational use of antibiotics. Current technology allows the rapid spread of knowledge without limitations and can help create programs through which students and doctors can easily obtain the latest information on this topic [46,47]. In our study, 5.7% of parents stated that they administered antibiotics without a pediatrician’s recommendation because the pediatrician has previously prescribed the same antibiotic for the same symptoms, and 2.2% stated that the reason for self-administration was a lack of time to visit the pediatrician or a lack of money to pay for treatment. A small percentage of parents, 3.7% and 2.5%, said that the reason was advice by a pharmacist or friend, respectively. This occurrence was significantly more often in parents with a lower educational level when compared with parents with a higher educational background. Our results are comparable with a study by Siddiqui et al. [48], where the most common reason described for self-administration of antibiotics was mentioned to be advice by a family member or pharmacy personnel or physician previously prescribing the same antibiotic [48].

Ninety-three percent of our parents knew that paracetamol and ibuprofen were not antibiotics, 80.7% knew that Panklavis an antibiotic, and 52.5% identified Pancefas an antibiotic. Parents with a higher education level had better knowledge of whether the offered medicine was an antibiotic. Although in our study parents were overall knowledgeable in regard to recognizing antibiotics, their administration during the illness of their children and their side effects, we noticed that 34.2% of parents believe that scientists can produce new antibiotics that could destroy resistant bacteria. This opinion is significantly (*p* < 0.001) more often shared by parents with a lower educational level when compared to parents with a higher educational background. These data show that a significant number of parents believe that scientists continuously discover new antimicrobial drugs. 

## 5. Conclusions

This study indicates that in the RS, parents recognize their pediatrician as their main source of information, and that they are pediatricians with rational antibiotic use and antibiotic resistance. Parents with a higher level of education have significantly better knowledge, attitudes and practices when it comes to the use of antibiotics. Pediatricians should be aware of the limitations of knowledge and the high possibility of inappropriate antibiotic use by parents of children, especially parents with a lower level of education. Therefore, this study concludes that simultaneous well-structured interventions for parents and pediatricians are needed to improve the consumption of antibiotics. Namely, campaigns through television and social networks presenting real facts about the dangers of irrational use of antibiotics for children’s health and the possible development of antimicrobial resistance can contribute to reducing the trend of self-medication and pressure on pediatricians by parents of sick children. On the other hand, professional education and training of pediatricians and empowering them with examples from practice, as well as encouraging close cooperation with pharmacists, can additionally help in solving the examined problem.

## 6. Limitations

This is the first study that was conducted to assess parental and pediatricians’ knowledge, attitudes and practice on antibiotic use among children in the RS. Nevertheless, there were some limitations of this study, and they were associated with the use of the convenience sample, which cannot be representative of the general population in the RS. Furthermore, the data were collected from parents who are attending health care centers or preschool institutions, and the overall higher educational level in the study sample may indicate possible bias. A great number of parents consisted of women, who had a higher level of education in comparison to men, and this could also indicate possible bias.

## Figures and Tables

**Table 1:** Demographic characteristics of the parents.

Variable	Data, Number (%) or Mean (SD)
Parental gender	
Male	357 (24.5)
Female	1102 (75.5)
Age, years	34.47 (6.15)
Minimum	16
Maximum	59
Area of living	
Urban	1201 (82.3)
Rural	258 (17.7)
Mother’s education	
Primary school	32 (2.2)
High school	629 (43.1)
College	78 (5.3)
University degree	720 (49.3)
Father’s education	
Primary school	27 (1.9)
High school	790 (54.1)
College	101 (6.9)
University degree	541 (37.1)
Family income	
Very low	17 (1.2)
Low	143 (9.8)
Average	1189 (81.5)
High	109 (7.5)
Very high	1 (0.1)
Single parents	
Yes	67 (4.6)
No	1392 (95.4)
Relationship with pediatrician	
Professional	930 (63.7)
Friendly	500 (34.3)
Familiar	29 (2.0)
Number of children	
0	10 (0.7)
1	471 (32.3)
2	697 (47.8)
3	236 (16.2)
4	39 (2.7)
5	6 (0.4)
Total	1459 (100)

SD—standard deviation.

**Table 2:** Demographic characteristics of the pediatricians and their knowledge about antibiotics and antimicrobial resistance.

Variable	Data, Number (%)
Gender	
Male	5 (27.8)
Female	13 (72.2)
Age, years	
36–45	4 (22.2)
46–55	2 (11.1)
56–65	11 (61.1)
>65	1 (5.6)
Years of service	
3–5	2 (11.1)
6–10	2 (11.1)
16–20	5 (27.8)
21–25	4 (22.2)
>25	5 (27.8)
I believe that I am contributing to the fight against antibiotic resistance	
Yes	18 (100.0)
No	0 (0.0)
I know what antibiotic resistance is	
Yes	18 (100.0)
No	0 (0.0)
I know what information I should give patients about the rational use of antibiotics and antibiotic resistance	
Yes	14 (77.8)
No	4 (22.2)
I have enough knowledge about how to use antibiotics properly in my practice	
Yes	13 (72.2)
No	5 (27.8)
Antibiotics are effective against colds and flu	
Yes	0 (0.0)
No	18 (100.0)
Taking antibiotics is associated with side effects or risks such as diarrhea, colitis, allergy	
Yes	18 (100.0)
No	0 (0.0)
Any person treated with antibiotics is at increased risk of developing an antibiotic-resistant infection	
Yes	17 (94.4)
No	1 (5.6)
Healthy people can be carriers of antibiotic-resistant bacteria	
Yes	18 (100.0)
No	0 (0.0)
Total	18 (100.0)

**Table 3:** Attitudes and practice of pediatricians about antibiotics and antimicrobial resistance.

Variable	Data,
	Number (%)
In the last 12 months, do you recall receiving any information about the importance of avoiding unnecessary antibiotic prescribing?	
Yes	11 (61.1)
No	5 (27.8)
I am not sure	2 (11.1)
What source(s) of information had the greatest impact on changing your understanding?	
Workplace	0 (0.0)
Through published guides	8 (44.4)
From colleagues	2 (11.1)
From a scientific organization	5 (27.8)
From social networks	0 (0.0)
Based on the information you received, have you changed your antibiotic prescribing practice?	
Yes	11 (61.1)
No	2 (11.1)
I am not sure	5 (27.8)
At what level do you think it is most effective to fight against antibiotic resistance?	
Action is needed at all levels	16 (88.9)
At the individual level (all healthcare workers)	6 (33.3)
EU/global level	6 (33.3)
Regional/national level	4 (22.2)
There is good promotion of the rational use of antibiotics and antibiotic resistance in the Republic of Srpska	
Yes	10 (55.5)
No	8 (44.5)
How often do you prescribe antibiotics?	
Every day	11 (61.1)
Weekly	7 (38.9)
I take antibiotic resistance into account when treating a patient	
Yes	12 (66.7)
No	6 (33.3)
How often was the fear of worsening the patient’s condition or the fear of complications the reason to prescribe an antibiotic?	
More than once a day	1 (5.6)
Less than once a week	2 (11.1)
Once a week or more	6 (33.3)
Never	9 (50.0)
How often have you prescribed an antibiotic because you were unsure of the diagnosis of an infection?	
Once a day	1 (5.6)
Rarely	11 (61.1)
Never	6 (33.3)
What strategies do you use to rationally prescribe antibiotics?	
Education of patients	18 (100.0)
Consultation with a new patient	2 (11.1)
Delayed prescribing	10 (55.6)
None	0 (0.0)
Total	18 (100.0)

**Table 4:** Main sources of information when deciding on the use of antibiotics with regard to level of education of parents.

Statement about the Main Source of Information		Primary and High School		College and University Degree		Total (*n* = 1459)		*p* (χ^2^)
		*n*	%	*n*	%	*n*	%	
Mothers	Doctors							
	No	7	1.1	16	2.0	23	1.6	0.149
	Yes	654	98.9	782	98.0	1436	98.4	
	Television							
	No	637	96.4	783	98.1	1420	97.3	0.039 *
	Yes	24	3.6	15	1.9	39	2.7	
	Friends							
	No	622	94.1	774	97.0	1396	95.7	0.007 *
	Yes	39	5.9	24	3.0	63	4.3	
	Total	661	45.3	798	54.7	1459	100.0	
Fathers	Doctors							
	No	14	1.7	9	1.4	23	1.6	0.635
	Yes	803	98.3	633	98.6	1436	98.4	
	Television							
	No	786	96.2	634	98.8	1420	97.3	0.003 *
	Yes	31	3.8	8	1.2	39	2.7	
	Friends							
	No	772	94.5	624	97.2	1396	95.7	0.012 *
	Yes	45	5.5	18	2.8	63	4.3	
	Total	817	56.0	642	44.0	1459	100.0	

χ^2^—Chi-square test; * statistically significant.

**Table 5:** Knowledge of parents about antibiotics.

Statement whether Offered Medicine is Antibiotic		Primary and High School		College and University Degree		Total (*n* = 1459)		*p* (χ^2^)
		*n*	%	*n*	%	*n*	%	
Mothers	Panklav							
	No	174	26.3	108	13.5	282	19.3	<0.001 *
	Yes	487	73.7	690	86.5	1177	80.7	
	Paracetamol							
	No	588	89.0	769	196.4	1357	93.0	<0.001 *
	Yes	73	11.0	29	3.6	102	7.0	
	Pancef							
	No	396	59.9	297	37.2	693	47.5	<0.001 *
	Yes	265	40.1	501	62.8	766	52.5	
	Ibuprofen							
	No	602	91.1	764	95.7	1366	93.6	<0.001 *
	Yes	59	8.9	34	4.3	93	6.4	
	Total	661	45.3	798	54.7	1459	100.0	
Fathers	Panklav							
	No	198	24.2	84	13.1	282	19.3	<0.001 *
	Yes	619	75.8	558	86.9	1177	80.7	
	Paracetamol							
	No	741	90.7	616	96.0	1357	93.0	<0.001 *
	Yes	76	9.3	26	4.0	102	7.0	
	Pancef							
	No	448	54.8	245	38.2	693	47.5	<0.001 *
	Yes	369	45.2	397	61.8	766	52.5	
	Ibuprofen							
	No	759	92.9	607	94.5	1366	93.6	0.201
	Yes	58	7.1	35	5.5	93	6.4	
	Total	817	56.0	642	44.0	1459	100.0	

χ^2^—Chi-square test; * statistically significant.

**Table 6:** Knowledge of parents about antibiotics.

Statement		Primary and High School		College and University Degree		Total (*n* = 1459)		*p* (χ^2^)
		*n*	%	*n*	%	*n*	%	
Mothers	An antibiotic should be given to every child who has a fever							
	Disagree	478	72.3	724	90.7	1202	82.3	<0.001 *
	Neutral	101	15.3	54	6.8	155	10.6	
	Agree	82	12.4	20	2.5	102	7.0	
	Scientists can easily produce new antibiotics that could destroy RB							
	Disagree	134	20.3	250	31.3	384	26.3	<0.001 *
	Neutral	261	39.5	315	39.5	576	39.5	
	Agree	266	40.2	233	29.2	499	34.2	
	Antibiotics can be harmful							
	Disagree	57	8.7	31	3.9	88	6.0	<0.001 *
	Neutral	145	21.9	136	17.0	281	19.3	
	Agree	459	69.4	631	79.1	1090	74.8	
Fathers	An antibiotic should be given to every child who has a fever							
	Disagree	626	76.6	576	89.8	1202	82.3	<0.001 *
	Neutral	112	13.7	43	6.7	155	10.6	
	Agree	79	9.7	23	3.6	102	7.0	
	Scientists can easily produce new antibiotics that could destroy RB							
	Disagree	195	23.9	189	29.5	384	26.3	0.032 *
	Neutral	326	39.9	250	38.9	576	39.5	
	Agree	296	36.2	203	31.6	499	34.2	
	Antibiotics can be harmful							
	Disagree	59	7.3	29	4.5	88	6.0	0.002 *
	Neutral	176	21.5	105	16.4	281	19.3	
	Agree	582	71.2	508	79.1	1090	74.8	

RB—resistant bacteria; χ^2^—Chi-square test; * statistically significant.

**Table 7:** Parental reasons for administering antibiotics without a pediatrician’s recommendation.

Statement		Primary and High School		College and University Degree		Total (*n* = 1459)		*p* (χ^2^)
		*n*	%	*n*	%	*n*	%	
Mothers	Lack of free time to visit a pediatrician or lack of enough money to pay for treatment							
	Never	629	96.6	776	98.9	1405	97.8	<0.001 *
	Frequently	12	1.8	6	0.8	18	1.3	
	Always	10	1.5	3	0.4	13	0.9	
	Pediatrician has previously prescribed the same antibiotic for the same symptoms							
	Never	566	91.4	742	96.6	1308	94.3	<0.001 *
	Frequently	20	3.2	16	2.1	36	2.6	
	Always	3	5.3	10	1.4	43	3.1	
	Pharmacist has recommended an antibiotic							
	Never	579	94.2	752	98.2	1331	96.4	<0.001*
	Frequently	16	2.6	11	1.4	27	2.0	
	Always	20	3.3	3	0.4	23	1.7	
	Friends/relatives recommended an antibiotic							
	Never	590	96.1	758	98.5	1348	97.5	0.001 *
	Frequently	10	1.6	7	0.9	17	1.2	
	Always	14	1.0	4	0.5	18	1.3	
Fathers	Lack of free time to visit a pediatrician or lack of enough money to pay for treatment							
	Never	778	97.3	627	98.6	1405	97.8	0.023 *
	Frequently	13	1.6	5	0.8	18	1.3	
	Always	9	1.1	4	0.6	13	0.9	
	Pediatrician has previously prescribed the same antibiotic for the same symptoms							
	Never	786	92.9	592	96.1	1308	94.3	0.031 *
	Frequently	22	2.9	14	2.3	36	2.6	
	Always	33	4.3	10	1.6	43	3.1	
	Pharmacist has recommended an antibiotic							
	Never	730	11.6	601	97.7	1331	96.4	0.036 *
	Frequently	20	2.6	7	1.1	27	2.0	
	Always	16	2.1	7	1.2	23	1.7	
	Friends/relatives recommended an antibiotic							
	Never	743	96.9	605	98.2	1348	97.5	0.007 *
	Frequently	12	1.6	5	0.8	11	0.8	
	Always	12	1.6	6	1.0	18	1.3	

χ^2^—Chi-square test; * statistically significant.
